# Semi-customized three-dimensional ultra-fine titanium meshes in guided bone regeneration for implant therapy in severe alveolar bone defect: a case report

**DOI:** 10.1186/s40729-024-00535-0

**Published:** 2024-03-29

**Authors:** Dae-Ho Park, Jong-Hun Jun, Seo-Hyoung Yun, Baek-Sun Choi, Joseph P. Fiorellini, Marco Tallarico, Kyung-Gyun Hwang, Chang-Joo Park

**Affiliations:** 1 Division of Oral and Maxillofacial Surgery, Department of Dentistry, College of Medicine, Hanyang University, 222 Wangsimni-ro, Seongdong-gu, Seoul, 04763 Republic of Korea; 2 Tissue Regeneration Institute, Osstem Implant Co. Ltd., Seoul, Republic of Korea; 3 Department of Periodontics, School of Dental Medicine, University of Pennsylvania, Philadelphia, PA USA; 4 Department of Medicine, Surgery, and Pharmacy, University of Sassari, Sassari, Italy

**Keywords:** Alveolar bone augmentation, Barrier membrane, Dental implant, Guided bone regeneration, Titanium mesh

## Abstract

This case report provides a detailed description of a simple and fast bone regeneration procedure using a semi-customized three-dimensional ultra-fine titanium mesh. A 50-year-old male with a severe vertical and horizontal bone defect in the anterior mandible underwent implant treatment in a staged approach. The autologous bone was combined with a xenograft, and the mixture was grafted to augment the bone defect and covered with semi-customized ultra-fine titanium meshes, which were selected among its various types according to size and configuration of the bone defect, directly connected and immobilized on the tenting screws with minimal shaping. In a postoperative 6 months re-entry surgery, the performed titanium meshes were removed, implants were placed, and a bone core biopsy was obtained that demonstrated satisfactory new bone formation. Finally, two months later, the definitive prosthesis was installed. This semi-customized ultra-fine titanium mesh could help an implant clinician obtain more predictable results in the guided bone regeneration (GBR).

## Background

Guided bone regeneration (GBR) is a technique used to augment lack of horizontal and/or vertical alveolar bone [1]. There are several important principles for success of GBR, including exclusion of epithelial tissue, maintenance of grafting space, stabilization of a fibrin clot, and undisturbed healing by tension-free wound closure [2–6]. To accomplish those principles, resorbable or non-resorbable barrier membranes should be incorporated into GBR procedures. A resorbable collagen membrane is easy to manipulate and requires no additional surgery for removal, but it is difficult to predict the exact degradation or resorption time and to maintain a larger grafting space without collapsing. On the contrary, a non-resorbable membrane requires additional surgery for removal, but generally shows a better result of bone regeneration, unless healing is interrupted by exposure [7].

Titanium mesh is a representative type of non-resorbable membrane that is characterized by inherent rigidity and produces satisfactory bone regeneration in large defects [7]. However, bone regeneration with titanium mesh could be complicated by exposure, which frequently results from sharp points and angles from its cutting, bending, and trimming to fit the titanium mesh to cover the grafted bone. Moreover, such grafts are vulnerable to postoperative infection [7, 8]. The term of “semi-customized” is used in this study since the titanium meshes is not completely customized using CAD/CAM. Instead, clinician could select from a range of stock meshes that were available in different size and configurations for each patient’s bone defects. In this case report, we describe a simple and fast GBR procedure that uses semi-customized ultra-fine titanium meshes for application to a large bone defect.

## Case presentation

### Patients and surgical procedure

**Fig. 1:**
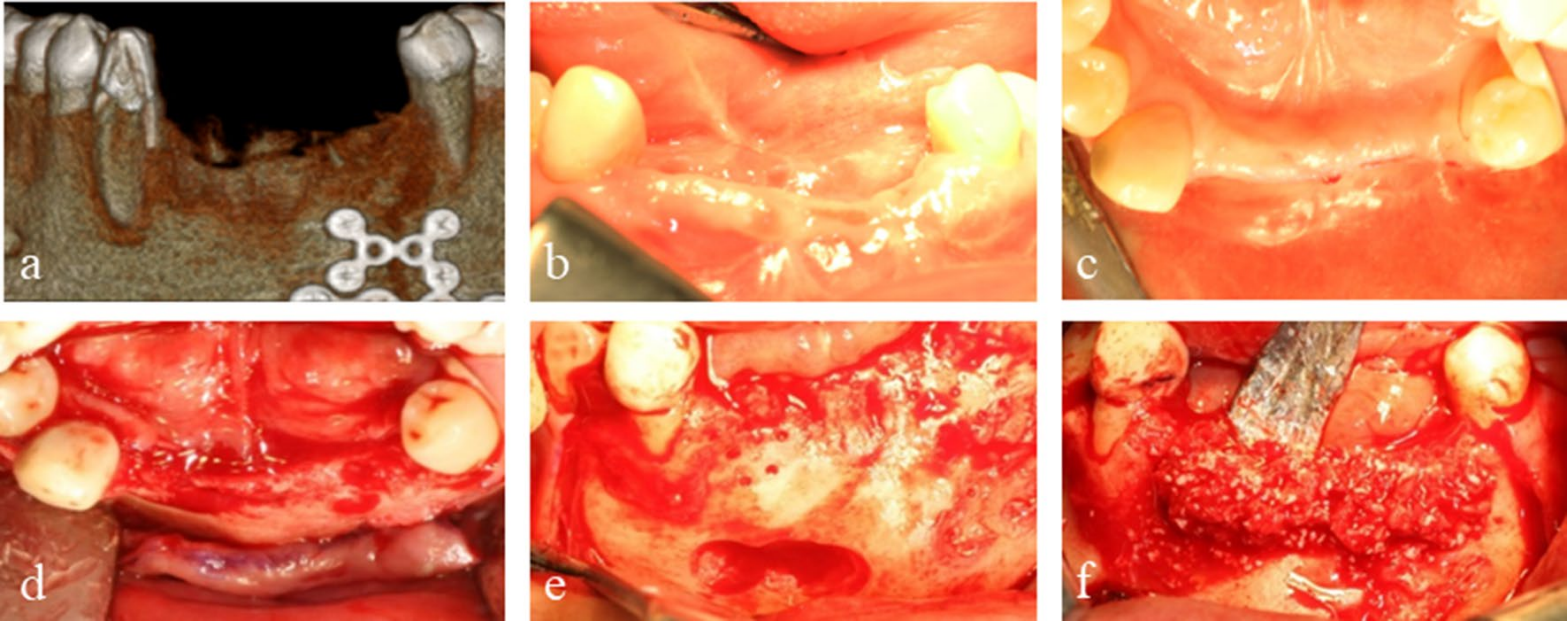
Preoperative and intraoperative intraoral images. **a** Preoperative 3D-reconstructed CBCT. **b** Preoperative intraoral view (buccal). **c** Preoperative intraoral view (occlusal). **d** Horizontal and **e** vertical defects shown clinically after the reflection of a mucoperiosteal flap during the surgery. **f** The graft material, a mixture of autologous bone and bovine xenograft, was gently placed to augment the large bone defect

A 50-year-old male with a combined vertical and horizontal alveolar bone defect in the anterior mandible due to previous facial trauma, was referred for implant treatment. His past medical history included open reduction and internal fixation of a compound mandibular fracture resulting from a traffic accident. The left mandibular lateral incisor and canine were avulsed with the alveolar bone fracture and the left mandibular central incisor and the right mandibular central incisor and lateral incisor were extracted during the fracture surgery. A crown fracture of the right mandibular canine was restored with post and core after a root canal treatment. In preoperative cone-beam computed tomography (CBCT) evaluation, the defect of the anterior mandibular area was measured to be approximately 7 mm vertically and 5 mm horizontally (Fig. 1a).

**Fig. 2:**
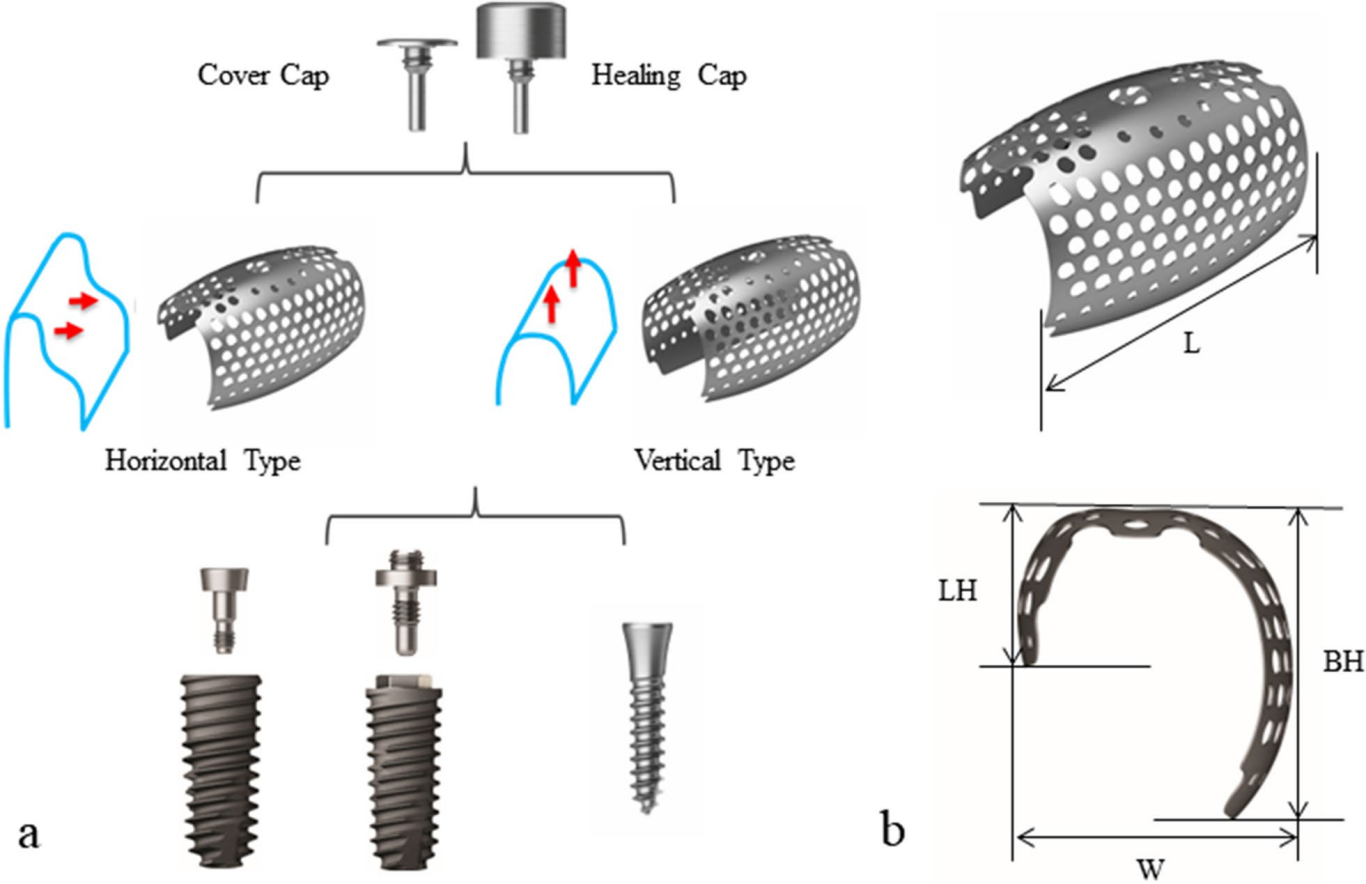
Types of semi-customized ultra-fine titanium meshes. **a** Horizontal and vertical types, which are connected to an implant fixture or tenting screw. **b** Divided into subsets with combinations of length (L; 10 and 20 mm), width (W; 9 and 11 mm), buccal height (BH; 7, 9, and 11 mm), and lingual height (LH; half or equivalent to BH) according to size and configuration of defects

**Fig. 3:**
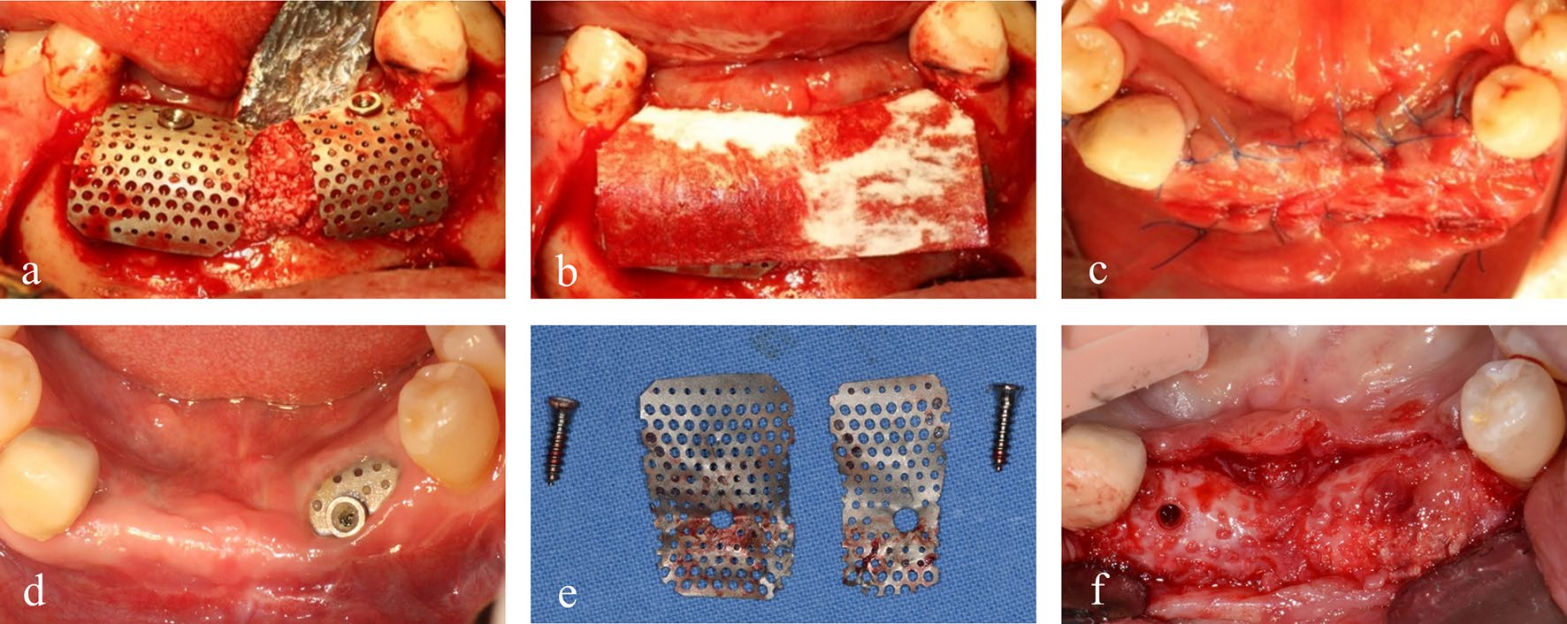
**a** Placement of the semi-customized ultra-fine meshes over the graft material. **b** The titanium meshes were overlaid with a resorbable collagen membrane. **c** Tension-free primary wound closure. **d** Exposure of the titanium mesh before the re-entry surgery. **e** Removal of tenting screws and titanium meshes. **f** Regenerated bone was observed in the location of titanium mesh removal

The patient provided informed consent, and staged bone augmentation was carried out for implant placement. After infiltration of 2% lidocaine with 1:100,000 epinephrine, a mid-crestal incision with two vertical incisions was performed from the left mandibular first premolar to the right first premolar, and a full-thickness mucoperiosteal flap was reflected. The plates and screws from the previous fracture surgery were removed. Two tenting screws were inserted in the optimal positions of the subsequent implants, and the size and configuration of the bone defect, which had been assessed preoperatively by CBCT, were confirmed clinically and carefully measured. The autologous bone, which was harvested from the location of the plates and screws and particulated by a specialized trephine drill (autobone collector, Osstem, Republic of Korea), was mixed with a xenograft (A-Oss, Osstem) in a 1:1 volume ratio and grafted to augment the vertical and horizontal bone defect around the tenting screws (Fig. 1b–f). Two semi-customized ultra-fine titanium meshes (OssBuilder, Osstem), as a horizontal type with dimensions of length 10 mm, width 9 mm, buccal height 11 mm, and lingual height 5.5 mm, were specifically chosen from various subsets available (Fig. 2). These titanium meshes were adjusted to cover the grafted area by minimal cutting and modeling and immobilized by cover caps. A resorbable collagen membrane (OssMem hard, Osstem) was placed over the titanium meshes to minimize premature thinning of the overlying gingiva. Finally, tension-free primary closure was performed with a releasing incision on the buccal periosteum (Fig. 3a–c). The patient received oral antibiotics and analgesics and was instructed to use a 0.12% chlorhexidine gluconate solution for 10 days.

### Results

Except for a small exposure of the semi-customized ultra-fine titanium mesh at the position of the left mandibular canine, there were no complications. The exposure size steadily increased up to approximately 5 mm in diameter. The tissues around the exposure were reddish in color but exhibited no adverse signs or inflammation. Six months later, a re-entry surgery was performed under local anesthesia and the titanium meshes were removed. Gross examination revealed regenerated bone, which was corticalized and lined with a thin pseudoperiosteum. However, in the area where the titanium mesh was exposed, a thicker pseudoperiosteum was present, intermingled with raw particles of xenograft. This pseudoperiosteum easily detached from the underlying regenerated bone during implant drilling (Fig. 3d–f).

**Fig. 4:**
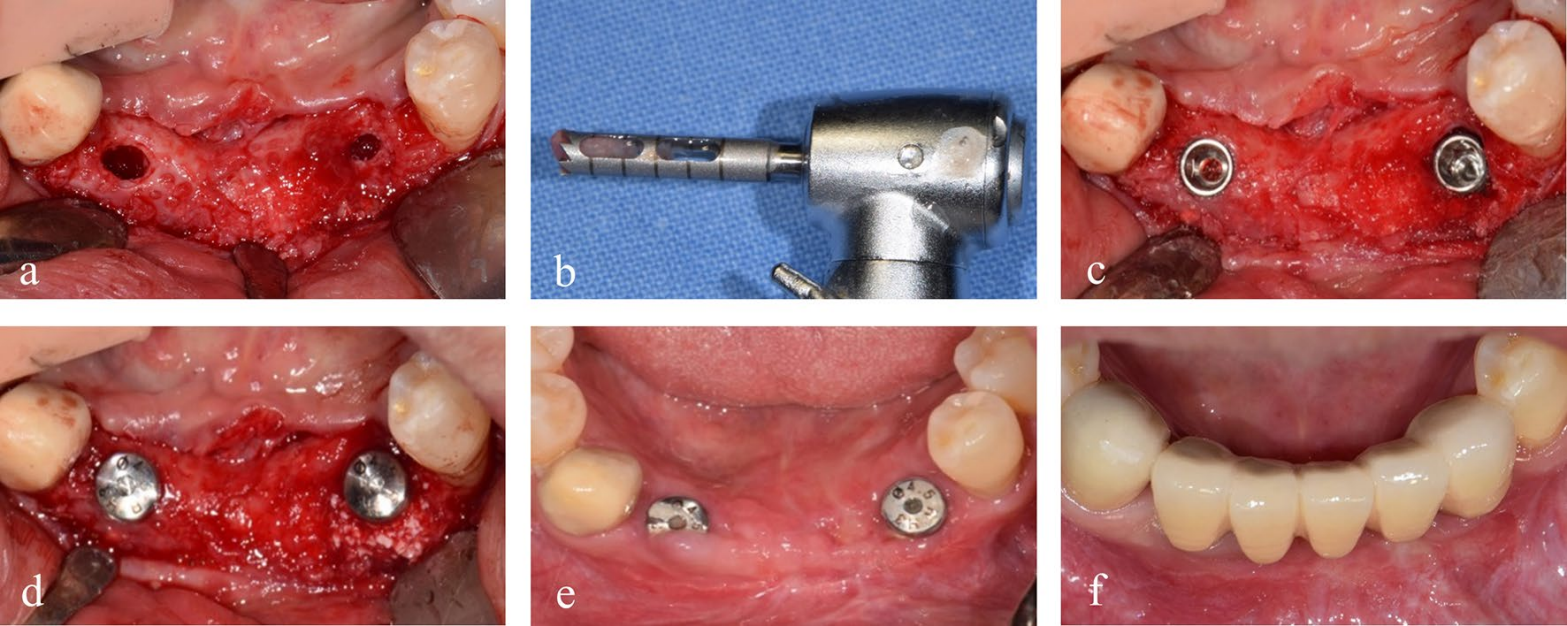
**a** A bone core was obtained near the tenting screw on the right side by a **b** trephine drill. **c** Two implant fixtures were placed. **d** An additional xenograft was placed on the left peri-implant dehiscence defect. **e** 2 months later, uneventful healing was observed around the healing abutments. **f** The definitive restoration was delivered

**Fig. 5:**
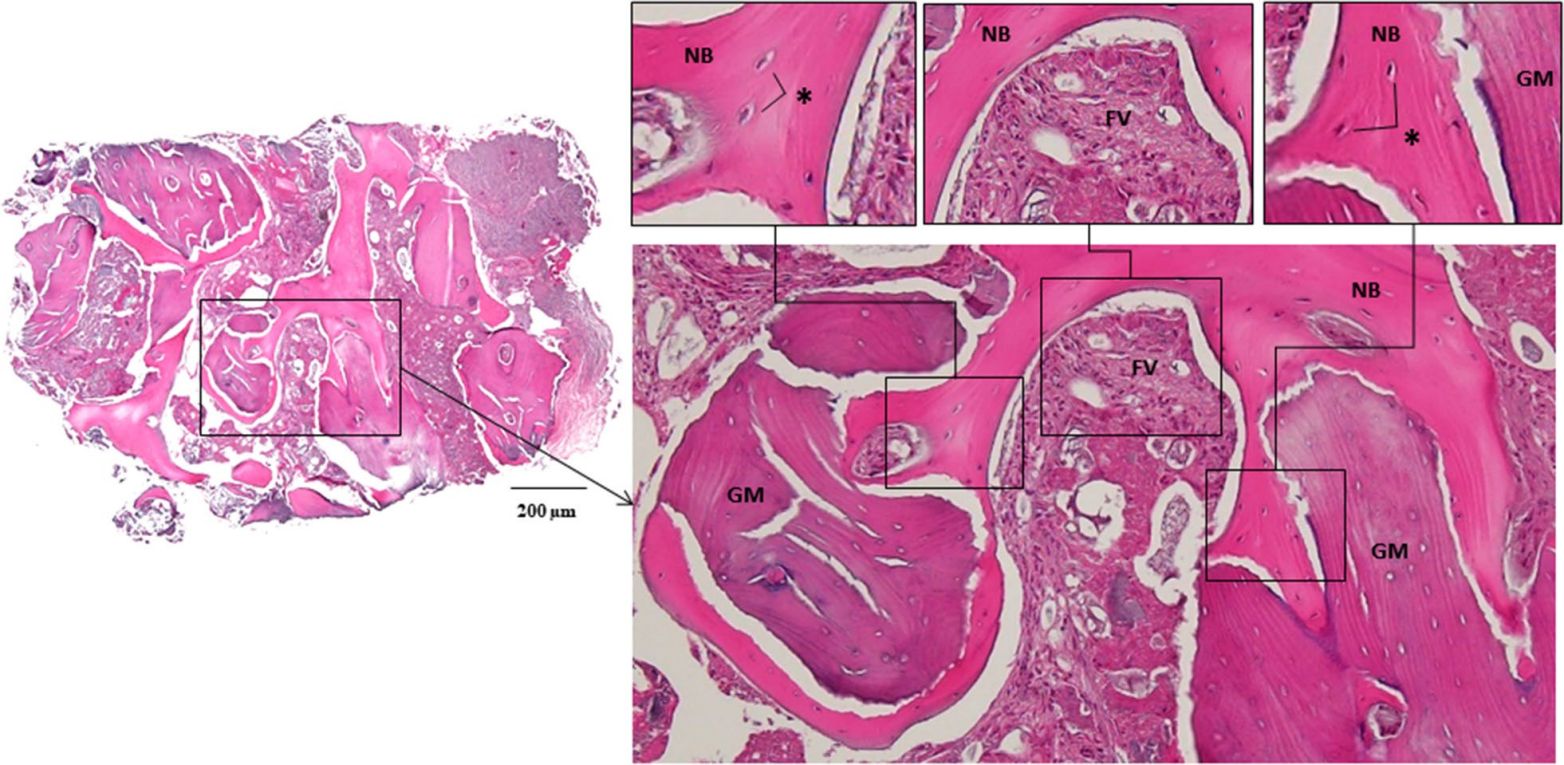
A histologic analysis of the bone core, showing the presence of the fibrovascular tissue (FV), residual graft material (GM), and the formation of new bone (NB). Arrows indicate the osteocytes in NB

**Fig. 6:**
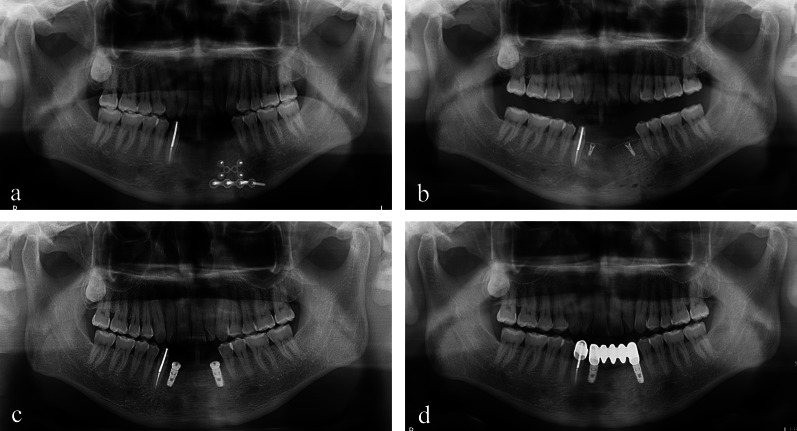
Serial panoramic views. **a** Preoperative. **b** GBR procedure. **c** Implant placement during re-entry surgery 6 months later. **d** Delivery of the definitive restoration after 2 months

**Fig. 7:**
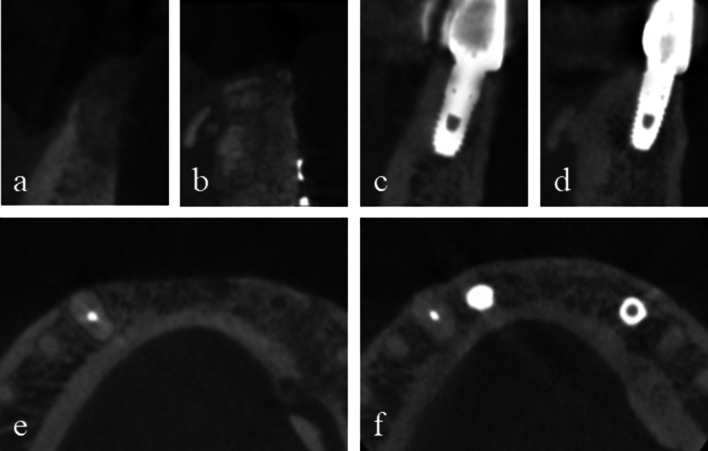
Serial CBCT views. **a**, **b** Preoperative cross-sectional slices of the right mandibular lateral incisor area and the left mandibular canine area. **c**, **d** Postoperative 6-year cross-sectional slices of implants corresponding to the right mandibular lateral incisor and the left mandibular canine. **e**, **d** Preoperative axial slice. **f** Postoperative 6-year axial slice.

Two 4.0 × 10 mm implant fixtures (TS III SOI, Osstem) were placed in the regenerated bone of the left mandibular canine and the right lateral incisor with a seating torque exceeding 30 Ncm. Two 5.0 × 3 mm healing abutments were then connected. Before implant drilling in the area corresponding to the left mandibular canine, a bone core was harvested for histological analysis using a 2.0-mm-diameter trephine drill (Fig. 4). The sections were stained with hematoxylin and eosin (H&E) and were observed using an optical microscope (Fig. 5). The areas of new bone, residual graft material and fibrovascular tissues were divided, and their relative percentages were measured. Two months later, impression taking and delivery of the definite zirconia restoration were performed. The postoperative panoramic radiographic revealed stable peri-implant bone level and notably, the patient expressed satisfaction with the final outcome (Figs. 6 & 7).

### Discussion

In cases of combined severe horizontal and vertical bone defects, bone grafting should be performed before implant placement. During this step-by-step treatment approach, the stability of the bone grafting space can be disturbed by routine events in the oral cavity, such as mastication and speech. Therefore, if a patient requires a substantial amount of bone graft, titanium mesh is considered the most suitable non-resorbable barrier membrane for maintaining the shape and volume of bone graft because, as a light metal, it is stiff and cost-effective [9–11]. Compared to the micropores of other barrier membranes, titanium mesh is unique in that it has macropores that do not selectively block or pass cells. Due to its biocompatibility and blood clot stabilization, titanium mesh has also been widely used in the field of oral and maxillofacial surgery.

Although flexible and smooth-textured, titanium mesh has a critical disadvantage of exposure, which is mostly not covered again by gingiva. The incidence of exposure associated with titanium mesh was reported to range from 16 to 34% [12]. Its stiffness is favorable for the maintenance of the grafting space, but it frequently causes thinning of the overlying mucosa during wound contraction in the healing period. Furthermore, using a titanium mesh as a barrier membrane is a very technique-sensitive procedure because it should be shaped and rigidly fixed. During this shaping procedure, the sharp points and angles caused by cutting, bending and trimming the titanium mesh could contribute to its exposure through the gingiva.

Different from a conventional titanium mesh plate, a semi-customized ultra-fine titanium mesh can be customized just by selecting the most proper one among its various types and subsets according to size and configuration of the bone defect. Thus, it can be used simply and quickly because customization allows minimal modification, eliminating sharp points and angles during routine shaping. Generally, as the semi-customized ultra-fine titanium mesh is chosen by assessing accurately the type, length, width, and height of the peri-implant bone defect by a periodontal probe or implant depth gauge and directly connected to the implant, bone grafting could be performed specifically and efficiently for the most optimal result. Moreover, its rigid fixation and stable immobilization are also achieved easily and rapidly. Considering that three dimensional printing of a patient-specific titanium mesh based on a preoperative CBCT dataset is expensive and requires complicated digital workflow [13], this semi-customized ultra-fine titanium mesh is cost-effective and ready-to-use, like a ready-made titanium mesh. If necessary, this semi-customized ultra-fine titanium mesh can be modified by minimal trimming and bending for better fit to a bone defect. Furthermore, the titanium mesh and its components used in this case were versatile, extending their utility to implants with internal and external connections made from other manufacturers.

The semi-customized three-dimensional ultra-fine titanium mesh is composed of grade 2 commercially pure titanium, with a thickness of 0.1 mm. It has three pores with different sizes; 1.0 mm around the implant, 0.6 mm on the extension and 0.5 mm on the sides of titanium mesh. With the 1.0 mm pores, appropriate blood supply and growth factor diffusion can be provided for the promotion of healing. The 0.6 mm pores also allow adequate blood supply and can prevent the bone graft material from migrating [8]. Additionally, semi-customized ultra-fine titanium mesh has 0.5 mm pores on its sides which are designed to preserve the mechanical rigidity during its shaping.

Autologous bone, the gold standard of bone grafting material due to its osteogenic effect, was harvested, particulated and used in the present case. Cortico-cancellous bone was used for the volume-maintaining effect of hard cortical bone [14, 15] and the pluripotential effect of loose cancellous bone [16]. Common intraoral sites for collecting autologous bone include the mandibular symphysis, maxillary tuberosity, ramus, tori, or exostoses [17]. In our case, the operation site was the anterior mandible area, therefore, autologous bone was harvested from the mandible symphysis with a small extension of the flap reflection, which did not require an additional surgical site. Reducing the number of surgical sites has the advantages of rapid recovery and fewer complications. In addition, a bovine xenograft, which showed little or no resorption during osteoconduction, was mixed into the autologous bone. In histomorphometric analysis, the removal of the semi-customized ultra-fine titanium meshes revealed successful bone regeneration lined with a thin pseudoperiosteum. Histologically, it was measured to be composed of 36.6% new bone, 36.0% residual graft material, and 27.4% fibrovascular tissue at the re-entry (Fig. 5).

However, in the area of titanium mesh exposure, the pseudoperiosteum was much thicker, and the quality of underlying regenerated bone was so poor that the regenerated bone could not resist implant drilling and broke off and an additional xenograft was necessary to cover the two exposed threads of implant top. A previous study also showed that it was possible to leave titanium mesh in place after exposure, although less regenerative bone was found in the exposed area [18]. The application of a resorbable collagen membrane over the titanium mesh, as in this case, is currently controversial. Our purpose was to promote cell occlusiveness and prevent premature thinning of the overlying gingiva by creating a clear separation between the compartments for both osseous and epithelial regeneration [7, 19].

## Conclusion

In conclusion, the present case demonstrated that GBR can be performed simply and quickly with a semi-customized ultra-fine titanium mesh. The semi-customized ultra-fine titanium mesh should be properly selected according to size and configuration of the bone defect and then connected and immobilized to the implant fixture or tenting screw. This semi-customized ultra-fine titanium mesh could help an implant clinician obtain more predictable results in the GBR for severe horizontal and vertical alveolar bone defects.

## Data Availability

Not applicable.

## Abbreviations

GBR: Guided bone regenerataion
CBCT: Cone-beam computed tomography
